# Recent Patents of Value to Dentists

**Published:** 1906-03

**Authors:** 


### RECENT PATENTS OF VALUE TO DENTISTS.

810611, Dental obtunder, Wesley F. Blasingame, Moultrie, Ga.
810712, Artificial denture, John M. Card, Olean, N. Y.
810713, Artificial denture, John M. Card, Olean, N. Y.
816842, Artificial denture, John M. Card, Olean, N. Y.
811370, Dental can, Wm. Brush, Weehawken, N. J.
811479, Dental compress, Nathan L. Burke, Benton Harbor, Michigan.
810954, Toothpick holder, Wilhelm Loewen, Glatz, Germany.

811442, Dental and surgical lamp, Clarence F. Rodgers, Conneaut, Ohio.
811848, Dental clamp, Edson L. Hutchinson, Honolulu, Hawaii.
811849, Tooth separator, Edson L. Hutchinson, Honolulu, Hawaii.
811864, Dental and surgical obtunder, Wm. L. Moore and E. L. Hutchinson, Honolulu, Hawaii.
811806, Mirror for surgical, dental, and like uses, Robert Walker, Newark, N. J.
812395, Portable dental furnace, Frederick S. Belyea, Brookline, Mass.
812328, Dental matrix, Wm. Crenshaw, Atlanta, Ga.
812567, Dental instrument holder, James W. Ivory, Philadelphia, Pa.

